# Rochester Hahnemannian Society

**Published:** 1888-05

**Authors:** W. H. Baker

**Affiliations:** Secretary


### THE ROCHESTER HAHNEMANNIAN SOCIETY.

The regular monthly meeting of the Rochester Hahnemannian Society was held Tuesday, March 20th, at the office of Dr. A. B. Carr, the President, Dr. R. C. Grant, in the chair.

After the regular order of business, paragraphs 105 to 115 inclusive of the *Organon* were read by Secretary, which was the investigation of the morbific power of drugs necessary for the cure of disease, and their primary and secondary action.

Dr. Biegler—There is one point that occurs to me in thinking over the good effects of our small doses, in our not getting the reaction spoken of in the sections read. Our patients recover without this reaction, and the mongrels get into a mess in consequence of large doses and frequent repetition, and when the reaction comes, they select another remedy as blindly as before and get into greater trouble, then fail and discard Homœopathy. I would like one of these gentlemen to see a case I saw this morning, and again to-night; they would have had a reaction and then made a mess of the case. The case is one of a child generally full of life and play. The symptoms were drowsiness, increasing for twenty-four hours. *Indisposed to move, lifeless and listless.* Fauces dry and looking dark, covered with cotton-like mucus. Right tonsil swollen and red. ℞ Bapt.$^{mm}$, 1 dose, at eleven A. M. To-night the child was full of play, with its usual life and activity, craving for food.

Dr. Schmitt—This reminds me how these gentlemen are prescribing. I read in a journal a paper that was read before the State Society. It was in regard to the use of Caulophyl., in preparing women for the lying-in state. Fifty women in a hospital were given the drug, and fifty did not receive any. The latter got along as well as the fifty who had Cauloph. The conclusion was, Cauloph. is no use in the lying-in state.

Dr. Carr—The sections read give us warning not to change the remedy, or repeat when these secondary symptoms come up. It has been the hardest lesson for me to learn to understand and control these secondary symptoms. A few days ago I gave Lach.$^{cm}$, one dose, for a Lachesis sore throat, and advised patient not to go to business. I was called in the evening to hurry and come, as my patient was worse, *he felt much worse*, had continued to grow worse all day. Pulse was five beats higher. Judging from his general appearance, tongue, etc., and as a number of symptoms that he had had were gone, I continued Sacch. lact., and the next day he was well.

Dr. Grant—There is another point: these gentlemen do not look at their books or pay any attention as to the drugs that follow each other, and will give drugs that are inimical to each other.

Dr. Biegler—In regard to the kind effects of the homœopathic dose, will illustrate by the case of a boy brought to me from Cincinnati. He had been sick since last September with what the doctor said was typhoid fever. The child suffered with a chronic diarrhœa. I did not see the child at first, but from the mother received indications for Arg-nit., which cured the diarrhœa in twenty-four hours. After a day or two the bowels moved naturally and have remained well since; but the boy was not well, and believing there was some element in the case that was not understood, I found out, on questioning the mother closely, that the boy had fallen out of an apple-tree a few weeks before he was taken sick, and had struck on his back. He was taken into the house insensible. Here was a case shattered by a fall. I gave Hyper. and cured the child, and with ten remedies, one dose of each of the MM potency, and which these scientific gentlemen could not do.

Dr. Schmitt—The latest humbugging and scientific practice is the treatment of the Crown Prince.

Dr. Baker then read a paper on Hydras-can.

Adjourned to meet at Dr. Biegler's office in one month.

W. H. Baker, M. D., *Secretary.*

---

### HYDRASTIS CANADENSIS.

W. H. Baker, M. D., Rochester, N. Y.

This is an indigenous plant, found growing in different parts of the United States and Canada, where the soil is damp and rich. It is known by various names, *i. e.*, ground raspberry, tumeric root, yellow puccoon, and golden seal, which is the most common. It was first known to the white settlers only through the Indian tribes, as they came in contact, with whom it was very popular as a medicine; so that the medical history of this drug dates back into the dim, traditional history of this country. It was used first by the botanic physicians; later the eclectics were not slow to avail themselves of the virtues of this plant. It is only through the hands of the homœopathic school that a remedy can reach complete development, and it was left for them to bring out the hidden virtues of this drug, by subjecting it to a physiological proving, as they have so many of the remedies of the materia medica.

MIND.—Disposed to be spiteful, disagreeable toward his friends, with desire to snub them. Spitefulness, with desire to knock things about generally. (Inclined to be out of humor, and angry, Ledum.) Disinclination to work, to apply himself (Caust., China, Kali b., Kobaltum, Nit. ac., Phytolac., Rumex, Spigelia, have similar condition). Forgetfulness while writing, cannot remember what he is reading or talking about. (Conium, Hyper., Lach., Lil. tig., Nux vom., Rhus tox., Sulph.) (Forgetful; in the middle of a speech, the most familiar words fail him—Baryta carb.) (Memory so weak does not even know his own room, Psorinum.) Vertex—Headache every other day, commencing at eleven A. M. (Rumex eleven P. M.) Sulph. antidotes the head symptoms. Eczema on margin of hair in front; worse coming from the cold into a warm room; oozing after washing. Severe frontal headache. Dull, heavy frontal headache over the eyes; severe cutting pains in the temples, and over eyes. Worse over the left; relieved by pressure. Dull frontal headache, with dull pain in the hypogastrium and small of the back.

Profuse lachrymation of the eyes, which smart and burn. The secretion from the mucous membranes is increased, and *thick, yellow, ropy, tenacious, mucous discharges* are characteristic of this drug.

Thick mucous discharges from both the eyes and ears. (Calc., Lyc., Merc., Phos., Puls., Sulph.)

Tickling, like a hair in the right nostril (left nostril, Kali bich.), (sneezing with frequent tickling and crawling in the nose, left nostril, Carbo veg.). The air feels cold in the nose. Continued flow of watery coryza; disappears in warm room; much aggravated in the open air. Thick, yellow, tenacious mucus from nose, more from posterior nares. Nosebleed, left nostril. (Right nostril Kali bich., Kali chlor.) with burning rawness, followed by itching. Coryza watery and excoriating, acrid (Ars, Lyc., Merc.), burning in the nose, *more the right nostril.* (Arum tri. has a corrosive excoriating, yellow discharge from nose, making the upper lip sore, *worse left nostril.*)

The tongue is coated white, or has a yellow stripe down the centre; swollen, and, like Merc., shows the imprints of the teeth. Tongue feels burned or scalded; vesicles form on the tip (Kali iod., Lyc., Nitr.); peppery taste in the mouth. If there be any secretion from the mouth and throat it will be the thick, tenacious mucus. Mercury and Chlorate of Potash are antidoted by this remedy, and after their abuse in stomititis and ulcers of the throat, in nursing women or weakly children, give this drug.

Scraping in the larynx.

In indigestion with loss of appetite, due to an atonic state of the stomach, study this remedy. When Lyc. seems to be indicated and does not give relief, this remedy will often give excellent results. Sour fluid eructations; *faintness at the stomach; great sense of sinking and goneness at the epigastrium, with violent and continued palpitation of the heart.* (Act. rac., Ars., Bapt., Phos., Sang., Murex, have this feeling of goneness in the stomach), (Sulph. has empty, gone, or faint feeling at eleven A. M., cannot wait for her dinner). Hydrastis has this difference, the goneness is accompanied with palpitation of the heart; goneness at eleven A. M. (Asafœt., Natrum carb.); acute, distressing, cutting pains in the stomach. In cancer of the stomach, when the patient vomits all she eats except milk and water, with emaciation and goneness; stomach actually sunken, weak, faint; bread and vegetables cause acidity; burning region of navel, with *goneness.* (Coloc., Petrol., *Phos.*, Sars., *Sepia*, Stram., have a feeling of emptiness or goneness in abdomen); loud rumbling, with dull aching in the hypogastrium and small of back, worse moving. Cutting pains in the abdomen, with heat faintness, better after passing flatus; fœtid flatus; constant sensation in both groins as if he had strained himself from taking a very long step; it is aggravated by touch, the clothes are uncomfortable; cutting pains extending from hypogastrium into testicles, with a dull, dragging pain in the groins; faintness after stool.

Stool is light-colored, soft, and acrid, sometimes greenish; torpor of the liver, with scanty, lumpy stools, covered with mucus; exhaustion from a light hemorrhoidal flow. In catarrh of bladder we have again the thick, ropy mucous sediment in the urine; urine smells decomposed, is increased and of neutral reaction. Gonorrhœa, second stage, with the characteristic thick, yellow discharge; debility; ulceration of the cervix and vagina; leucorrhœa is yellow, thick, tenacious, and ropy. Pruritus vulvæ, with the characteristic leucorrhœa. Uterine diseases, with sympathetic affections of the digestive organs; rawness, soreness, and burning in the chest; bronchitis of old, exhausted people; thick, yellow, tenacious, stringy sputa. In phthisis, to relieve the goneness in stomach, emaciation, loss of appetite; dry, harsh cough from tickling in larynx; violent and long-continued palpitation of the heart in the morning.

Muscles are greatly weakened; atony. Great soreness and harshness of the muscles of the neck.

Painful, tired feeling across small of back, waist, and lower limbs. Pain in left side of neck to shoulder, relieved by laying the hand on it.

Sore pain in small of back and region of kidneys. Pain as if tired in all the limbs; tired and ache with coryza. Shifting pains in right arm and leg, then left leg; stinging rheumatic pains in the elbows, forearms, and knees; knee ache. Sharp pain from right hip joint to knee, making it impossible to stand or bear one's weight. While walking, *severe pain* in outer part of left knee, causing limping; at the same time, pressure in left shoulder. Aching in outer part of left knee while sitting, worse when walking. Aching in sole of left foot; no relief from change of position. Sulph. antidotes the sciatic pains.

Sleep. Awakened by backache, and dull pains in navel and hypogastric region. Troublesome, worrying dreams. Difficulty in awaking.

Chill morning or evening: Chilliness, especially in the back or thighs, with aching; pulse slow. Heat in flushes. Great heat of the whole body. Constant dull burning pains all the evening. Fœtid perspiration of the scrotum. Erysipelatoid rash on the face, neck, palms, joints of fingers, and wrist, with maddening, burning heat; later, skin exfoliates; pains worse at night. Itching tingling of the eruption in variola; face swollen; throat raw; pustules dark; great prostration. The eclectic school prescribe this drug in debilitated conditions, and it is to them what Quinine is to the allopath. In cancer, where there is great debility, cutting pains like knives, and the cancer is hard, adherent, skin mottled and puckered. In great debility, following gastric, bilious, and typhoid forms of fever, it will be found useful.

